# Shaping of microbial phenotypes by trade-offs

**DOI:** 10.1038/s41467-024-48591-9

**Published:** 2024-05-18

**Authors:** Manlu Zhu, Xiongfeng Dai

**Affiliations:** State Key Laboratory of Green Pesticide, School of Life Sciences, Central China Normal University, Wuhan, PR China

**Keywords:** Bacteriology, Microbial ecology, Bacterial physiology, Bacterial systems biology

## Abstract

Growth rate maximization is an important fitness strategy for microbes. However, the wide distribution of slow-growing oligotrophic microbes in ecosystems suggests that rapid growth is often not favored across ecological environments. In many circumstances, there exist trade-offs between growth and other important traits (e.g., adaptability and survival) due to physiological and proteome constraints. Investments on alternative traits could compromise growth rate and microbes need to adopt bet-hedging strategies to improve fitness in fluctuating environments. Here we review the mechanistic role of trade-offs in controlling bacterial growth and further highlight its ecological implications in driving the emergences of many important ecological phenomena such as co-existence, population heterogeneity and oligotrophic/copiotrophic lifestyles.

## Abstract (web summary)

Trade-offs play a key role in controlling bacterial growth and shaping microbial phenotypes, which further drives the emergence of ecologically relevant phenomena including co-existence, population heterogeneity and oligotrophic/copiotrophic lifestyles.

## Introduction

Rapid growth is a fundamental property of microbes and is, in principle, advantageous for microbial fitness. Bacterial cells are capable of tightly coordinating macromolecule biosynthesis (mass accumulation) with cell cycle progression (number increase) to ensure rapid proliferation^1–6^. Protein synthesis lies at the core of bacterial growth as protein accounts for over half of the biomass and its synthesis consumes ~70% of the cellular energy budget^3,5–8^. Furthermore, mRNA transcription is globally coordinated with protein translation under various conditions^9–11^. For the model bacterium *Escherichia coli*, ribosome synthesis could be maximized in nutrient broth to support a rapid growth rate of ~20 min per doubling^5,12–15^. However, the nutrient status in ecological niches of bacteria is often highly fluctuating, leading to the so-called ‘feast and famine cycle’^14,16–19^. For enteric bacteria living in mammalian gut, feast-like conditions occur when abundant amino acids and carbohydrates are available after the meals of the host while famine-like conditions occur when bacteria are released into the sewer^14,20,21^. Similar scenarios also occur for soil bacteria living in plant rhizosphere, which feed on the root exudates of the host plant^22–25^ and also for marine bacteria living in either oligotrophic open sea or eutrophic coastal area^16,26–29^. Therefore, it is important to elucidate how microbes coordinate cell growth with gene expression to adapt to different nutrient environments.

Recent quantitative studies have revealed the fundamental role of proteome resource allocation in governing microbial growth control^3,5,6,8,30–34^. To maintain optimal growth statuses, bacteria attempt to balance their proteome investments in various functional sectors according to the nutrient conditions^6,14,35–38^. In a rich medium where nutrient sources are abundant, bacteria could save the proteome resource of anabolic proteins and further maximize ribosome synthesis to achieve fast growth^8,13,15,39^. When shifting to a minimal medium, bacteria employ (p)ppGpp-DksA to inhibit ribosome synthesis and devote more resources into anabolic proteins to fulfill the supply of in vivo amino acid flux^5,8,19,40,41^, which inevitably results in slower growth. Furthermore, cAMP-CRP allows bacteria to fine-tune the proteome allocation between catabolic and anabolic proteins to adapt to different carbon or nitrogen sources^31,42^. In this sense, bacteria employ sophisticated molecular strategies to efficiently modulate proteome allocation so that optimal growth statuses could be achieved in various nutrient conditions^6^. A three-sector proteome allocation model could quantitatively describe the tight relation between gene expression and growth rate with only a few phenomenological parameters^6,30,31^.

In order to further accelerate growth, microbes including bacteria and yeast attempt to balance energy biogenesis and biomass synthesis^43–46^. A long-standing puzzle associated with microbial energy biogenesis is the overflow metabolism, in which fast-growing cells (bacteria, yeasts and cancer cells) preferably use the seemingly low-efficient fermentation pathway instead of respiratory pathway to generate ATP during growth under aerobic conditions, thus secreting large amounts of fermentation products such as acetate and lactate^47–49^. Recent studies have shown that overflow metabolism results from the strategy of optimal proteome resource allocation for achieving rapid growth^44,50^. Although fermentation has a lower ATP production efficiency than respiration, it costs fewer enzymes and requires a lower proteome investment per ATP produced than respiratory pathway. In such case, employment of fermentation pathways for energy biogenesis allows microbes to save more proteome resources for ribosome synthesis to achieve faster growth under favorable conditions^44,46,47^. A similar logic applies to another puzzle of prokaryotic energy generation: the prevalence of Entner–Doudoroff (ED) pathway in many aerobes^51^ considering that ED pathway produces only one ATP per glucose—half of EMP pathway. This puzzle could again be rationalized by the fact that ED pathway costs much less enzymes to achieve the same glucose conversion rate than the EMP pathway, thus facilitating rapid growth^43^.

Although growth rate maximization is an important fitness strategy for bacterial cells, a seemingly paradox lies at the extremely large variations in the growth capacities of bacteria across phylogeny^52,53^. The maximal growth capacities of bacteria across species vary substantially with the shortest generation time ranging from 10 min (e.g. *Vibrio natriegens*)^54^ to days (e.g. *Mycobacterium tuberculosis*, Mtb and marine oligotrophic bacterium Pelagibacterales SAR11)^55,56^. In light of this fact, microbial ecologists conventionally divide bacteria into fast-growing copiotrophic bacteria (*r-*strategist) and slow-growing oligotrophic bacteria (*k*-strategist)^56–58^. Given that our ecosystems are often dominated by slow-growing oligotrophic bacteria^25,27,28,58–61^, e.g. the SAR11 bacterium that dominates the ocean ecosystem^56^, it is clear that rapid growth is often not the favorable trait to be selected across ecological environments. In addition, some clinically pathogens such as Mtb grow slowly and the slow growth phenotype is often associated with enhanced antibiotic tolerance, host adaptability and could further facilitate long-term latent infections and transmissions of pathogens^62–67^, posing the necessity of identifying the evolutionary driving force of slow growth phenotypes. Understanding the emergence of the diverse growth phenotypes of microbes is of fundamental importance for microbiology.

Recent studies have shown that a substantial fraction of proteome in bacteria could serve other important physiological objectives (e.g. adaptability and survival)^14,68–71^. These alternative objectives are often implemented at the cost of reduced growth rate as they drive proteome resources away from supporting biomass growth, leading to trade-offs^70^. Here we overview recent progress of trade-offs between growth and other physiological traits such as adaptability and survival, focusing on the mechanistic origins and physiological effects, and furthermore, highlighting their ecological implications in driving the emergence of phenotype diversity. We first introduce the trait of microbial adaptability to changing nutrient environments and its mechanistic link with proteome reserve. We then elaborate the trade-off mechanism between growth and adaptability across microbial species and its ecological implications in causing co-existence and phenotypic heterogeneity. We further discuss the trade-off relation between microbial growth and another core trait of fitness, survival and stress tolerance. We finally discuss how these fundamental trade-off principles could lead to the emergence of two major trophic phenotypes of microbes in our ecosystem: oligotrophic lifestyles versus copiotrophic lifestyles.

## Proteome reserve promotes adaption to nutrient transition

### Amino acid downshift

**Fig. 1:**
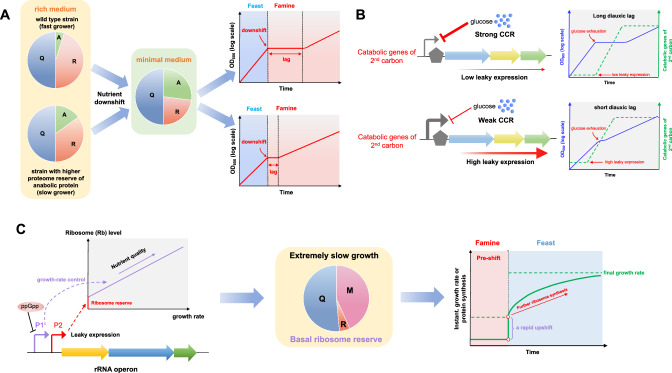
Proteome reserve from leaky expressions promotes adaptability to nutrient fluctuation. **A** During nutrient downshift from rich medium to minimal medium, bacterial cells have to re-allocate the proteome resource from ribosomes (R) to anabolic proteins (A). strains harboring a higher level of proteome reserve of anabolic proteins (A) could more quickly adapt to downshift with a shorter lag time than wild type strain. The Q sector denotes a growth-rate independent proteome sector for metabolic maintenance. **B** During carbon diauxic shift, strains of tight carbon catabolic repression (CCR) have low leaky expression for the catabolic genes of the 2nd carbon, and thus displaying a long diauxic lag. In contrast, strains of weak CCR have high leaky expression of related catabolic genes and could also trigger the induction of related catabolic genes before glucose exhaustion. As a result, strains of weak CCR display short or even no diauxic lag. **C** The control of rRNA synthesis contains two behaviors: a growth-rate dependent control mediated by (p)ppGpp on P1 promoter and a constitutive expression behavior from P2 promoter. The leaky expression of P2 promoter enables bacteria to maintain a basal ribosome reserve (R) in its proteome during extremely slow growth. The ribosome reserve allows bacteria to quickly initiate an upshift program during nutrient upshift from famine to feast conditions before making more ribosomes to further increase growth rate.

Since nutrient conditions are often highly fluctuating in nature, the adaptability to nutrient transition is crucial for microbial fitness^14,19,68,72,73^. The growth transition from good to poor nutrients is referred to as nutrient downshift while the opposite direction is referred to as nutrient upshift^74^. Upon a sudden nutrient downshift event, bacteria enter into a lag phase before fully adapting to the poor nutrient due to the time required for proteome re-allocation to meet the metabolic demand^68,73,74^. During the nutrient downshift from amino acid-supplemented rich medium to minimal medium, namely amino acid (AA) downshift, *E. coli* cells have to synthesize enough anabolic enzymes to supply the amino acid flux at the cost of downregulating ribosome synthesis^19,35^. A very recent study has shown that *E. coli* maintains a basal level of anabolic enzymes even when growing in nutrient broth^14^. The leaky expression of biosynthetic enzymes, namely proteome reserve, is actually crucial for the timely adaption of bacteria to sudden AA downshifts as it prevents the cells from being completely depleted of internal amino acid pools during downshift^14^. Slow-growing *E. coli* cells with increased basal levels of (p)ppGpp, harboring larger proteome reserve of anabolic proteins in rich medium, display much shorter lags during AA downshift and thus becomes a faster ‘switcher’ (Fig. 1A)^19^.

### Carbon diauxie

Carbon diauxic shift is a special case of nutrient transition that first described by Jacques Monod, whereby bacteria consume all of a preferred carbon source (e.g. glucose) before utilizing the secondary carbon source, resulting in diauxic lags^75,76^. The diauxie phenomenon, also referred to as ‘glucose effect’, mainly results from the inhibitory effect of glucose on the transport and catabolism of the 2^nd^ carbon via inducer exclusion^77^. Being similar to the case of AA downshift, the length of diauxic lag is tightly related to the leaky expression of the catabolic genes of the 2^nd^ carbon source. For example, the glucose-lactose diauxic lag of *E. coli* could completely disappear if the leaky expression of *lac* operon is stimulated by addition of exogeneous cAMP^78,79^. In contrast, imposing proteome constraint by expressing non-required proteins could limit the expression of catabolic operons of the 2^nd^ carbon and further prolong the diauxic lag^69^. A similar notion is also applicable to budding yeast. Compared with laboratory *Saccharomyces cerevisiae*, many natural isolates of *S. cerevisiae* and some other *Saccharomyces* species such as *S. bayanus* display much shorter or no diauxic lags during glucose-galactose shift due to a stronger leaky expression of the GAL gene (Fig. 1B)^80–83^. In addition, the induction of GAL genes could be bimodal for particular mixtures of glucose and galactose and for particular strains, leading to the emergence of two subpopulations including fast switcher (short diauxic lag) and slow switcher (long diauxic lag)^80,82,84^. Such a strategy of weakened carbon catabolic repression (CCR) is proposed to shift the yeast from is a ‘specialist’ to a ‘generalist’ in order to better adapt to changing environments^80,84^.

Mechanistically, both cAMP-CRP and (p)ppGpp regulate carbon diauxie of bacteria. cAMP-CRP promotes the emergence of carbon diauxic growth. CRP-cAMP activates the expression of glucose transporter (PtsG), enhancing the uptake of glucose and its conversion to glucose-6-phosphate, which then increases the dephosphorylated IIA^Glc^ and further prevents lactose uptake by inhibiting LacY activity, namely inducer exclusion^79^. (p)ppGpp-mediated stringent response maintains transcription-translation coupling during carbon transitions, further ensuring the timely expression of the catabolic genes of the 2^nd^ carbon source and facilitate the adaption of bacteria to the 2^nd^ carbon^19^. As a result, the stringent-response deficient *relA-null* strain exhibits a longer diauxic lag.

### Nutrient upshift

The strategy of proteome reserve is also crucial for bacteria during adaptation to nutrient upshift^85,86^. During nutrient upshift events, bacteria accelerate cell growth via upregulating ribosome synthesis and downregulating non-required metabolic proteins^74^. The P1 promoter of *rrn* operon is regulated by (p)ppGpp to achieve growth-rate dependent ribosome synthesis^87^. However, it has recently been found that *E. coli* and yeast cells maintain a ribosome reserve during slow growth conditions^32,88–90^, which comes from the leaky expression of rRNA genes (e.g. constitutive activity of *rrn* P2 promoter in *E. coli*)^87^. The ribosome reserve, being maintained in inactive states via either translation initiation inhibition or ribosome hibernation^91–93^, could be activated immediately during nutrient upshift to help microbes quickly resume protein synthesis and cell growth. In such case, microbes could significantly save the time required for de novo biosynthesis of more ribosomes (Fig. 1C)^85,86^.

Collectively, in both favorable and harsh nutrient environments, microbial cells manage to maintain a seemingly useless proteome reserve (either metabolic proteins or ribosomes). The proteome reserve, resulting from the leaky expression of related genes, is actually important for microbial cells to prepare for future changing environments and is thus advantageous for microbial fitness in fluctuating environments.

## Trade-off between growth and adaptability

Although rapid growth and rapid adaption to nutrient fluctuations could in principle both promote microbial fitness, it is difficult to simultaneously optimize both traits by bacteria due to conflicts of resource allocation. For example, the proteome reserve of anabolic proteins in rich medium could limit the cellular ‘budget’ for ribosome synthesis, thus comprising the maximal growth capacity of bacteria^14^ (Fig. 1A). Artificial (p)ppGpp induction in rich medium, although favors faster adaption to AA downshift via increasing the proteome reserve of anabolic proteins, concurrently reduces cell growth by inhibiting ribosome synthesis^19^. In one word, the improvement of adaptability often occurs at the expense of reduced growth rate, resulting in trade-offs. Similarly, for those *Saccharomyce* variants with short or no diauxic lags, they exhibit lower growth rates in glucose medium than the laboratory *S. cerevisiae* strains with strong diauxic phenotype due to the cost of increased leaky expression of GAL genes^81,83^. A recent work has quantitatively established a negative relation between growth rate and diauxic lag for *E. coli* during transition from various glycolytic carbons to the gluconeogenic carbon, acetate^68^. They found that cells growing slowly on poor glycolytic carbons (e.g. mannose) require much shorter lags than cells growing rapidly on good carbons (e.g. glucose) during acetate downshift as slow growth enhances acetate catabolism such as glyoxylate shunt pathway. In addition to the trade-off between adaptability and growth, recent work has shown two other forms of trade-offs during glycolytic-gluconeogenic carbon transition that intrinsically rooted in metabolism and physiology^94^. Firstly, trade-off exists between lags to glycolysis and gluconeogenesis due to biochemical constraints of flux sensing. During shift between glycolytic and gluconeogenic carbons, metabolite levels could quickly collapse to their equilibrium so that cells could be unable to sense the proper direction of metabolic flux. Bacteria could alleviate this problem by selecting a preferred direction of regulation at the expense of increasing lag times in the opposite direction^94^. Species specialized in glycolysis (e.g. *E. coli*) have short lags during shift to glycolytic carbon but long lags during shift to gluconeogenic carbon while species specialized in gluconeogenesis (e.g. *P. aeruginosa*) follow the opposite behavior^94^. This notion of preferential directionality in central carbon metabolism (glycolysis or gluconeogenesis) has further been confirmed by the Cordero lab using 186 marine heterotrophic bacterial strains and 135 carbon sources^95^. Secondly, attempts of optimizing both directions (e.g. increasing the abundances of gluconeogenic enzymes in glycolytic growth or glycolytic enzymes in gluconeogenic growth) reduce both lag times simultaneously but at the expense of increasing futile cycling and energetic cost, resulting in trade-off between lags and futile cycling that could affect bacterial survival during stress^94^.

### Co-existence of different phenotypes

**Fig. 2:**
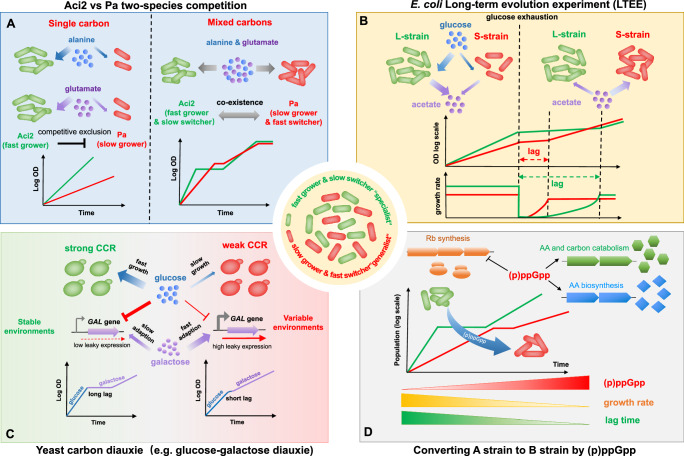
Growth-adaptability trade-off leads to co-existence and phenotypic heterogeneity. Two growth phenotypes could emerge in the population or microbial community: the ‘specialist’ population (fast grower & slow switcher) and the ‘generalist’ population (slow grower & fast switcher). **A** The co-existence of two bacterial species in the presence of multiple resources: *Acinetobacter species (Aci2)* and *Pseudomonas aurantiaca (Pa)*. When alanine or glutamate is present as the sole carbon source, Aci2 could competitively exclude Pa due to its higher growth rates. However, these two strains could co-exist when both alanine and glutamate are available as Pa could more quickly adapt to the alanine-glutamate diauxic shift with a much shorter lag than Aci2. **B** Two subpopulations co-exist in the long-term evolution experiment (LTEE) of *E. coli:* the L-strain and S-strain. The L-strain grows faster than S-strain in glucose medium and could secret a higher amount of fermentation products, acetate, into the medium. However, S-strain could adapt more quickly to the growth transition to acetate at the cost of reduced growth rate in glucose medium. As a result, although growing more slowly than L-strain in glucose medium, S-strain could gain a fitness advantage during transition to acetate after the exhaustion of glucose. **C** During the carbon diauxic growth of budding yeast, stochastic gene expression generates two subpopulations: subpopulation A of strong carbon catabolite repression (CCR) could grow fast at glucose medium but requires a long lag phase to adapt to glucose-galactose diauxic shift; subpopulation B of weak CCR could grow slowly in glucose medium but adapt more quickly to the diauxic shift with a short or no lag. **D** (p)ppGpp simultaneously regulates bacterial growth and the adaptability to nutrient fluctuations. (p)ppGpp inhibits ribosome (Rb) synthesis but activates amino acid (AA) biosynthesis as well as some AA and carbon catabolism processes. As a result, (p)ppGpp reduces bacterial growth but meanwhile promotes the bacterial adaptation to various types of nutrient downshift. Therefore, (p)ppGpp acts as a key regulator that could convert the bacteria from fast grower & slow switcher to slow grower & fast switcher.

The trade-off between growth and adaptability is crucial in shaping microbial phenotypes and could often lead to the co-existence of different growth phenotypes (illustrated in Fig. 2). In a recent work by Bloxham et al., the authors investigated the coexistence of two species including *Acinetobacter* species (Aci2) and *Pseudomonas aurantiaca* (Pa)^96^. They found that Aci2 could competitively exclude Pa on either alanine or glutamate due to its faster growth on single carbon source. However, Pa has a much shorter lag than Aci2 during alanine-glutamate diauxic growth. Therefore, the ‘fast grower’ (Aci2) and the ‘fast switcher’ (Pa) could coexist when both alanine and glutamate are present. In this sense, the trade-off between growth and adaptability could allow slow-growing microbes to coexist with fast-growing microbes in multi-resource environments (Fig. 2A). Furthermore, Basan group has recently investigated the coexistence of different phenotypes in the *E. coli* long-term evolution experiment (LTEE)^97^. They identified two key phenotypes associated with LTEE: L-strain and S-strain (Fig. 2B). The L-strain is a fermentation specialist that grows more rapidly on glucose and could secret more fermentation products, acetate. In contrast, S-strain could switch more quickly to acetate utilization after glucose exhaustion, albeit at the cost of a lower growth rate on glucose. Therefore, co-existence again emerges between a ‘fast grower’ (L-strain) and a ‘fast switcher’ (S-strain) in the microecosystem of LTEE. It is fascinating that the co-existence in LTEE could robustly persist over thousands of generations of evolution without being replaced by fitter strains, which suggests that trade-off here is fundamental and at least is not easily overcome by minor tweaks in metabolism. Trade-off can thus result in evolutionarily stable coexistence of different phenotypes, which cannot be invaded and replaced by fitter strains, further resulting in evolutionarily stable ecosystems.

### Bet-hedging via phenotypic heterogeneity

The trade-off between growth and adaptability poses challenges for microbes to adapt to an unpredictable environment. Phenotypic heterogeneity is an effective bet-hedging strategy for microbes to deal with the growth-adaptability trade-off. For example, during diauxic shift of *S. cerevisiae*, stochastic gene expression causes bimodality induction of the catabolic genes of the secondary carbon^80,82^, generating two subpopulations (Fig. 2C): a ‘fast grower’ (‘specialist’) subpopulation, with a stringent CCR, that displays a high growth rate in stable environments; a ‘fast switcher’ (‘generalist’) subpopulation, with a weak CCR, that could more quickly adapt to carbon transition at the cost of reduced growth rate in glucose due to high leaky gene expression. A similar phenomenon has been observed for *Lactococcus lactis* during glucose-cellobiose diauxie^98^. Therefore, phenotypic heterogeneity allows microbial population to balance growth and adaptability to maintain fitness in both stable and variable environments, achieving the objective of bet-hedging^84^. The emergence of phenotypic heterogeneity is found to be associated with metabolic heterogeneity during environmental changes^99^. The strategy of evolving phenotypic heterogeneity could have some specific fitness advantages over the simple strategy of adopting proteome re-allocation by a homogeneous population in highly fluctuating environments. In the former case, some subpopulations are capable of quickly adapting to the new environments with short lags, thus saving the time required for proteome re-allocation that could lead to long lags.

### The effect of (p)ppGpp

In addition to CCR, (p)ppGpp signaling is also tightly related to the trade-off between growth and adaptability. In rich medium, (p)ppGpp induction inhibits bacterial growth but accelerate the bacterial adaption to AA downshift via triggering a global resource re-allocation from ribosome synthesis to anabolic biosynthesis^19^. Moreover, in minimal medium, (p)ppGpp induction could activate the catabolism of certain amino acids such as alanine and arginine, further shorten the lag of *E. coli* during carbon downshift (from glucose to alanine) and nitrogen downshift (from NH_4_Cl to alanine or arginine)^100^. Therefore, (p)ppGpp induction could convert the bacteria from a fast-grower & slow switcher to a fast-switcher & slow grower (Fig. 2D).

## Trade-off between growth and survival

### Growth-survival trade-off

To thrive in nature, bacterial cells must rapidly proliferate under favorable conditions but persist during stress. Therefore, survivability during stress is also a key trait that determines bacterial fitness, and thus bacteria adopt sophisticated molecular strategies to balance growth and survival^101^. The (p)ppGpp-mediated stringent response and RpoS-mediated general stress response, the two major signaling pathways involved in stress response in many bacterial species, are largely activated during stress such as nutrient deprivation while the levels of (p)ppGpp and RpoS are relatively low during exponential phase to avoid their inhibitory effects on cell growth^102–104^. Nevertheless, strong trade-offs between growth and survival do exist in bacteria in many circumstances. For example, clinically isolated pathogens often exhibit a slow growth phenotype which is proposed to facilitate the adaption and survival inside host^62–66^. Recent studies by Gerland group investigated the effect of growth rate on the survival of *E. coli* during nutrient starvation^105,106^. They find that the death rate of *E. coli* cells during nutrient starvation is negatively correlated with the growth rate of cells before entering into starvation as slow growth results in decreased maintenance rates of cells. Basan group further shows that a growth rate-dependent proteome response, especially on envelop proteins, contributes to the promotion of survival by slow growth^107^. A striking growth-survival trade-off, resulting from proteome resource competition, has recently been reported for the gram-positive model bacterium *Bacillus subtilis*^70^. The authors find that the resource allocation strategy of *B. subtilis* does not lead to growth maximization on various carbons sources. Knockout of a master regulator gene, *spo0A*, triggers a proteome re-allocation from survival & stress response pathway to biosynthesis pathways and further accelerates cell growth, however, at the cost of compromised bacterial survivability after nutrient deprivation due to a decreased stress adaptability.

**Fig. 3:**
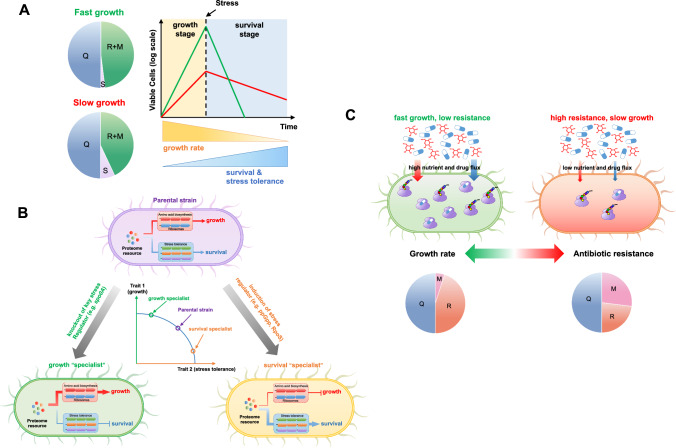
Growth-survival trade-off. **A** Slow growth could boost the survival and stress tolerance of bacteria. During slow growth conditions such as poor nutrients, bacteria could have a higher proteome investment on stress response (S), thus facilitating the long-term survival during sudden stress conditions. R+ M denotes the sum of growth rate dependent ribosomes (R) and metabolic sector (M). **B** Trade-off between growth and survival due to proteome allocation constraints. Growth and survival traits have proteome allocation conflicts and bacteria often need to balance the proteome investment on these two traits according to their environmental conditions. Knockout of some key stress regulators (e.g. *spo0A* in *B. subtilis*) favors growth at the cost of compromised survival, generating a growth ‘specialist’. In contrast, induction of certain stress regulators (e.g. ppGpp or RpoS protein) could favor survivability at the cost of reduced growth rate, generating a survival ‘specialist’. **C** An example of trade-off between growth and drug resistance against streptomycin for *E. coli*. Evolution favors the emergence of streptomycin resistant strains with compromised nutrient and drug uptake (right) so that the in vivo levels of both the drug and its ribosome targets decrease. As a result, resistance occurs at the cost of reduced drug-free growth rate due to decreased resource allocation towards ribosome synthesis (R sector) compared with native strain (left).

Mechanistically, the enhanced survivability during slow growth might attribute to an elevated stress response (Fig. 3A). The intracellular levels of key stress regulators, e.g. ppGpp and RpoS, are often growth rate dependent and increase significantly during slow growth^102,108–111^. Therefore, the leaky expressions of various stress responsive genes could also increase during slow growth and further facilitate bacterial survival under harsh environments^112^. In support of this picture, artificial induction of ppGpp or RpoS reduces cell growth but strongly enhances the stress tolerance of *E. coli* by triggering proteome re-allocations from biosynthesis to stress response^100,109,113^. In the case of *B. subtilis*, the positive effect of Spo0A on stress response could also be enlarged during slow growth due to an increased phosphorylation status of Spo0A under poor conditions^114^, imposing a stronger proteome burden on cell growth of *B. subtilis* in poor nutrients. As a result, the growth rate advantage of *spo0A*-null strain over wild type strain becomes even stronger on poor carbons^70^.

A similar example of growth-survival trade-off has been observed in the case of chemotaxis and motility, which are important for bacteria to survive in nutrient-limited ecological environments^115^. The chemotaxis and motility process of *E. coli* are positively regulated by CRP-cAMP and are thus upregulated on poor carbons^116^. Under carbon-limited conditions, a significant proteome resource is devoted to flagellar synthesis at the cost of reduced growth rates so that bacteria could become motile to search for more nutrient sources^37,44^. As a result, mutants devoid of flagellar synthesis gain a significant growth advantage over the parental strain in laboratory medium, especially on poor carbon sources^116^. Interestingly, Gude et al. has recently found that the growth-migration trade-off could also lead to co-existence between a fast-growing & slow-moving population and a fast-moving & slow-growing population^117^. They found that either of the two population could outcompete the other when low in relative abundance by active segregation and spatial exclusion within the patch. Such a type of inversion of the competitive hierarchy promotes the emergence of co-existence of the two populations and could, in principle, also promote phenotypic diversity in ecological niches.

The trade-off between growth and survival/stress response is not limited to bacteria but also found in eukaryotic microbes like yeast. A strikingly positive relation between division rate and death rate has been established in *Schizosaccharomyces pombe*^118^. Moreover, the expression of stress responsive genes is also negatively correlated with growth rate in *S. cerevisiae*, and thus slow growth also enhances the stress resistance of budding yeast^119^. Interestingly, trade-off between growth and mating also exists in *S. cerevisiae* due to the cost of gene expression^120^. Mutant devoid of mating gains a growth advantage during asexual stage while strains of increased mating efficiency mediated by GPA1 results in a growth disadvantage^120^.

Collectively, the fundamental growth-survival trade-off emerges as a result of conflicts of resource allocations. This principle could be taken advantage by microbial cells to convert their phenotype for surviving under various environmental conditions. For example, perturbing key signaling pathways (e.g. (p)ppGpp, RpoS, Spo0A) could favor either the growth trait or the survival trait by driving proteome resource towards either biosynthesis pathways or survival & stress response pathways (Fig. 3B)^70,100,110,113,121^.

### Antibiotic tolerance and resistance

A widely concerned topic of bacterial survival is antibiotic tolerance and resistance, which are indicated by MDK (minimal duration for killing) and MIC (minimal inhibitory concentration) of bacteria during antibiotic treatment, respectively^122^. On one hand, it has long been proposed that slow growth could enhance drug tolerance. For example, slow-growing bacterial populations, associated with lower speeds of cell wall assembly and DNA replication, have higher tolerances (slower killing rate by antibiotics) than the fast-growing populations against β-lactams and fluoroquinolones treatment^122,123^. Tolerance could be enhanced by both inherited slow growth (e.g. slow growers such as Mtb) and non-inherited slow growth (e.g. due to poor nutrients)^122^. Increased drug tolerance could further favor the emergence of drug resistance^124,125^. Drug tolerance associated with slow growth phenotype has been observed for not only Mtb but also other pathogens such as *Pseudomonas aeruginosa* and *Staphylococcus aureus*^62–64,66,125^. A reversion of the slow growth phenotype, either by modulating key signaling pathways or adaptive evolution, could restore the antibiotic susceptibility and compromise the bacterial fitness inside host^62,66^. On the other hand, the development of antibiotic resistance is often associated with a fitness cost such as reduced drug-free growth rates^126^. The trade-off between resistance and growth could be due to several reasons: (1) because antibiotics target essential biological processes (e.g. translation, transcription and metabolism), resistance mutations could compromise the normal structures and functions of related macromolecules such as ribosome, RNA polymerase or metabolic enzymes and further reduce cell growth^126–128^; (2) resistance mediated by overexpression of resistance proteins such as efflux pumps or enzymes (e.g. beta-lactamase encoded in plasmids) imposes proteome burden and energy cost on bacteria^126,129^; (3) In a recent study focusing on evolution of antibiotic resistance against streptomycin^130^, the authors found that evolution favored the emergence of resistance mutants with attenuated antibiotic and nutrient uptake, which led to the reduction of both in vivo drug concentration and ribosome targets. Therefore, the cost of growth in such case is due to decreased nutrient uptake and ribosome synthesis (Fig. 3C).

### Bet-hedging strategies to balance growth and survival

The fundamental growth-survival trade-off poses a challenge to microbial fitness as both of these two traits are crucial to sustain the bacterial population in natural niches, and therefore, bacteria sometimes manage to adopt bet-hedging strategies via generating phenotypic heterogeneity^131^. For example, *B. subtilis* initiates sporulation during unfavorable conditions such as starvation, however, only in a fraction of the population^114,132^. The rest cells could maintain growth with nutrients generated from cannibalism or autolysis^70,133^. Such a bet-hedging strategy allows the population to persist if the harsh environment continues or to quickly resume growth when the environments become favorable again. Starved *E. coli* population also adopts a bet-hedging strategy by evolving phenotypic heterogeneity when encountering new nutrient resources in order to break the fundamental trade-off between growth and survival^134^. The majority of cells could quickly resume growth after starvation with short lag times. Meanwhile, a small subpopulation with extreme long lags is retained and exhibits strong tolerance to environmental stressors such as antibiotics.

## Oligotrophic versus copiotrophic lifestyles

Our current understanding of microbial physiology is primarily based on a few fast-growing model organisms such as *E. coli* and *S. cerevisiae*. According to the fundamental notion of *r-/k-* strategy, bacteria are conventionally divided into two classes^53,57–59,135,136^: the fast-growing copiotrophs (*r*-strategist) thriving in environments with high nutrient opportunities; slow-growing oligotrophs (*k*-strategist) living in nutrient-limited environments. The classification of copiotroph and oligotroph, although might be over-simplified, pinpoints to an important reality that our ecosystems are mainly dominated by slow-growing bacteria^25,28,58–61^. Perhaps the most well-known example lies at the oligotrophic bacterium SAR11^56,137^ and *Prochlorococcus*^138,139^, which are among the smallest but most abundant microorganisms on earth and constitute nearly half of all planktonic cells in ocean. Compared with common copiotrophic bacteria, the growth of oligotrophic bacteria such as SAR11 and *Prochlorococcus* are much slower (days per doubling). Furthermore, they have streamlined genomes (~1.5 Mb) and very small cell sizes (~0.1 μm^3^)^56,137–139^.

### Growth efficiency and stress resistance

Direct comparisons between oligotrophs and copiotrophs highlight the intriguing trade-offs between growth rate and other traits such as growth efficiency^26,52,60,140–142^. It has been proposed that high-resource environments could favor faster growth but more energetically wasteful lifestyle (e.g. fermentation) due to increased competition while nutrient-limited environments favor relatively slow growth but more energy-efficient lifestyle (e.g. respiration)^44,141^. Although growing much more slowly, oligotrophic bacteria exhibit higher growth efficiency than copiotrophic bacteria^26,52,60^, especially in nutrient-limited environments as their high-affinity but low-specificity nutrient transport systems allow simultaneous uptake of mixed substrates^26–28,140^. The high growth efficiency of oligotrophic bacteria is associated with a low maintenance energy which could result from a minimization of the costs of various energy-consuming processes^136,140,142^. For example, the high-affinity, low-specificity transport systems enable oligotrophic bacteria to economically utilize multiple substrates under oligotrophic conditions with a relatively low amount of energy-intensive ABC transport systems^27,136^. Oligotrophic bacteria also tend to be non-motile^136,143^ and their genome streamlining properties^53,144^could further save the energy cost associated with macromolecular biosynthesis.

Strikingly, oligotrophic bacteria like *Sphingopyxis alaskensis* also display higher inherent stress resistance/tolerance than common copiotrophic bacteria against the treatment of hydrogen peroxide, high temperature, ethanol and UV during exponential growing^26,27,136,145^. Moreover, they could also sustain for a long time with no apparent loss of viability during nutrient starvation^26,146^. Many possibilities exist regarding the inherent stress resistance: bacteria have a constitutively high proteome investment on stress response; possession of some special stress protective mechanisms or/and the extremely slow growth itself simply results in stronger survivability.

### Constitutive gene expression and fast adaption

**Fig. 4:**
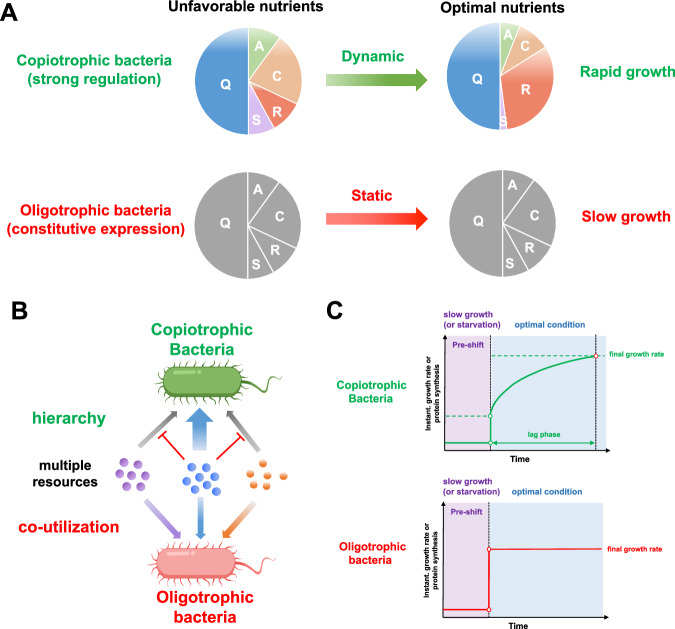
The oligotrophic versus copiotrophic strategies. **A** Proposed proteome allocation strategies for copiotrophic and oligotrophic bacteria. During transition from unfavorable nutrients to optimal nutrients, the strong transcription regulation in copiotrophic bacteria enables efficient dynamic proteome re-allocation among various sectors including catabolic proteins (C), anabolic proteins (A), ribosomes (R) and stress response proteins (S). As a result, ribosome synthesis could be maximized in optimal conditions to support fast growth. In contrast, the gene expressions of oligotrophic bacteria are largely constitutive, resulting in a static proteome allocation that limits ribosome synthesis and cell growth. **B** In the presence of multiple resources, the strong transcription regulation in copiotrophic bacteria such as catabolite repression leads to hierarchical utilization of substrates. In contrast, the constitutive expression strategy could enable oligotrophic bacteria to simultaneously utilize multiple substrates. **C** During nutrient upshift from unfavorable condition (poor nutrients or starvation) to optimal condition, copiotrophic bacteria need to re-allocate the proteome resource for maximizing ribosome synthesis to reach the final growth rate and thus require a lag time. Instead, oligotrophic bacteria adopt constitutive expression strategy and thus could reach the final growth rate immediately after a nutrient upshift process.

Compared with copiotrophic bacteria, oligotrophic bacteria exhibit a highly distinct feature of gene regulation: a significant reduction of transcription regulation with fewer sigma factors and two-component systems^53,56,143^. As a result, gene expressions in oligotrophic bacteria are highly constitutive and lack of growth-rate dependent control^143^. For example, oligotrophic bacteria, SAR11 and SAR92 are lack of growth control of ribosome synthesis^147^, a core mechanism employed by copiotrophic bacteria to achieve rapid growth in rich medium^111^. A series of global ‘omics’ studies have shown that oligotrophic bacteria exhibit weak alterations in transcriptome and proteome during changing growth conditions such as nitrogen limitation, sulfur limitation, glucose limitation as well as transition from exponential to stationary phase^143,145,148–150^. Transcriptome analysis suggests that only 0.1% of protein-encoding genes appear to be under transcriptional control in SAR11^149^. The constitutive gene expression pattern is not limit to bacteria and has also been found in methanogenic, oligotrophic archaeon *Methanococcus maripaludis*^151^. The abundances of various core proteome sectors such as translation machinery, catabolic proteins and anabolic proteins remain almost invariant across growth conditions for *M. maripaludis*. Instead, its modulation of the overall translation rate could be achieved by regulating ribosome activity rather than ribosome numbers^151^. Collectively, copiotrophic bacteria and oligotrophic bacteria follow distinct gene regulation strategies. A strong transcription regulation, e.g. mediated by (p)ppGpp and cAMP, enables copiotrophic bacteria to dynamically modulate the proteome allocation and achieve rapid growth via maximizing ribosome synthesis during favorable conditions^5,6,13^. In contrast, the constitutive gene expression pattern leads to a nearly static resource allocation strategy in oligotrophic bacteria and further limit the ribosome synthesis and maximal growth capacity (illustrated in Fig. 4A).

As described above for copiotrophic bacteria such as *E. coli*, the proteome reserve from the leaky expression (constitutive activity) of the promoters of metabolic proteins and ribosomes (Fig. 1), although limits growth rate, converts bacteria from ‘specialists’ to ‘generalists’ to efficiently utilize multiple resources with short or no delay. Due to the lack of transcription regulation, the constitutive expression strategy is much more prevalent in oligotrophic bacteria than copiotrophic bacteria^143^. In consistent with the constitutive expression strategy, oligotrophic bacteria could simultaneously utilize multiple substrates (Fig. 4B) and display no growth lags upon nutrient transition (Fig. 4C)^26,136^, allowing them to make full use of the limited nutrient resources available without any delays that could otherwise be associated with transcription reprograming process observed in copiotrophic bacteria^143^.

### ‘Oligotrophic state’ in copiotrophic bacteria

Although copiotrophic bacteria differ significantly from oligotrophic bacteria in the gene regulation strategy and lifestyle, it should be noted that sometimes an ‘oligotrophic state’ strategy is still retained in those fast-growing copiotrophic bacteria. For example, it has recently been found that a very small subpopulation of non-sporulating *B. subtilis* cells could enter into an extreme slow-growing ‘oligotrophic state’ during long-term starvation, which facilitates long-term survival and enables cells to become highly tolerant to stress and antibiotic treatment^152^. Moreover, the small fraction of slow-growing persister cells in the populations of *E. coli* and many clinical pathogens might also be viewed as a special case of oligotrophic lifestyle^122,153,154^. In this sense, copiotrophic bacteria still maintain a minimal set of oligotrophic strategies (e.g. proteome reserve strategy) as a bet-hedging approach to adapt to variable environments.

## Future perspectives and conclusion

Understanding the emergence of diversity of microbial growth phenotypes across species and environments is a central topic of microbiology. Our review here highlights the importance of trade-off principles in shaping the growth phenotypes of microbial cells across species and environments. It has recently been suggested that trade-off lies at the core of determining the qualities of different nutrient substrates^155^. Rather than reflecting fundamental biochemical or biophysical limitations, nutrient quality is largely a self-determined, plastic property that reflects the safety, reliability, and profitability of different ecological environments for a particular species and could be quickly adapted by evolution if necessary^155^. Microbial cells could employ nutrients as a signal to infer information about their environments based on their experiences in natural niches and further implement a trade-off decision of fast growth versus preparation for changing environments. Accordingly, bacteria could sense low-quality carbon sources as a signal of environmental deterioration so they activate the expressions of many proteins related to adaptability and stress response by ppGpp and cAMP signaling as a strategy to prepare for the changing conditions^155^. Therefore, trade-off between growth and adaptability/survival in proteome allocation could thus set the qualities of different carbon sources and result in the simple bacterial growth laws^31,156^. From this view, trade-offs in combination with ecological conditions could be the root cause of different growth phenotypes. We propose here that achieving rapid growth is just one aspect of concerns of microbial cells that does not have a higher priority over other physiological traits like adaptation and survival. Those alternative physiological traits could affect growth rates at which bacteria have evolved to grow, as much as growth rates affect them. This point is vividly illustrated by coexistence in the LTEE, where evolution pushes S-strain to slow down its growth rate and the slow growth phenotype further facilitates this strain to occupy the ecological niche. Trade-offs can thus be selected for and be the root cause of slow growth rates. This notion is perhaps further reinforced by the fact of the wide distribution of slow-growing oligotrophic bacteria in our ecosystems, considering that oligotrophic bacteria often display better performances on alternative traits such as long-term survival than the fast-growing copiotrophic bacteria.

**Fig. 5:**
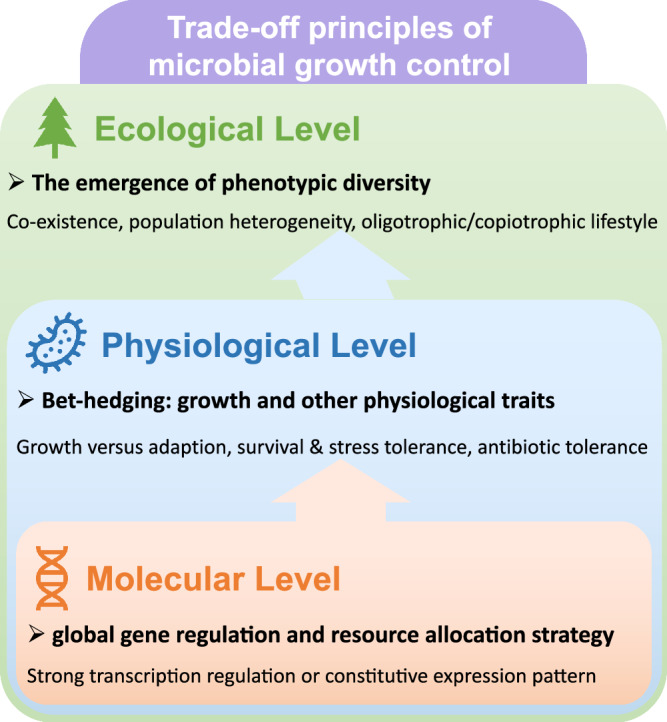
Trade-off principles of microbial growth control links three levels of microbiological disciplines. At molecular level, it is tightly related to the global gene regulation and resource allocation strategy of microbial cells. At physiological level, it is related to the bet-hedging strategy of microbial cells to balance growth and other key physiological traits such as adaptability, survival and stress tolerance as well as antibiotic tolerance. At ecology level, it is closely related to the emergency of phenotypic diversity that associated with many ecological phenomena such as co-existence of different phenotypes, population heterogeneity and the occurrence of oligotrophic/copiotrophic lifestyles.

From a general perspective, the trade-off principles in growth control discussed here directly link three key levels of microbiological disciplines (Fig. 5). At molecular level, it is closely related to the fundamental design principle of gene regulation and resource allocation strategy in bacteria. At physiological level, it connects microbial growth with many other important physiological traits such as survival and adaption, stress and antibiotic tolerance. It is ultimately related to the ecological level with important implications in the emergences of various ecological phenomena such as co-existence, population heterogeneity and oligotrophic/copiotrophic lifestyles.

Our current understanding of bacterial growth control is still largely limited to a few species. The proteome resource allocation strategy across conditions remains to be explored for those slow-growing oligotrophic species including extremophiles and archaea. It remains a grand challenge to identify key trade-off principles that drive the emergence of distinct growth phenotypes across species and across different ecological environments. To this end, it could be plausible to mimic certain natural environments of bacterial species in the laboratory and further interchange the living environments of different species to see how it could affect the evolution of growth and other related phenotypes. In addition, large interspecies variations in growth phenotypes emerge even among different natural isolates of the same species^81,157^. It is therefore important to investigate their distinct strategies of resource allocation and, furthermore, the underlying trade-off driving force. An ultimate challenge is whether we could quantitatively predict the evolutionary route of growth phenotypes from a long-term evolutionary scale. In another direction, trade-off principles shape the growth phenotype of many clinically related pathogens, further playing a key role in the evolvement of virulence and antibiotic tolerance. Mechanistic insights into the trade-off principles would guide the selection of proper antimicrobial drugs as well as development of novel antimicrobial therapies that further compromise the fitness of pathogens.
